# 2523. Outcomes by Resistance Phenotype and Genotype among Baseline Pathogen in Patients with Complicated Urinary Tract Infection (cUTI) in the Phase 3 CERTAIN-1 Study

**DOI:** 10.1093/ofid/ofad500.2141

**Published:** 2023-11-27

**Authors:** Greg Moeck, Leanne Gasink, Mary Beth Dorr, Hongzi Chen, Leah Woosley, Rodrigo E Mendes, Florian Wagenlehner, Tim Henkel, Paul McGovern

**Affiliations:** Venatorx Pharmaceuticals, Malvern, Pennsylvania; LBG Consulting, Saint Davids, Pennsylvania; Venatorx, Malvern, Pennsylvania; Venatorx Pharmaceuticals, Malvern, Pennsylvania; JMI Laboratories, North Liberty, Iowa; JMI Laboratories, North Liberty, Iowa; Justuf Liebeg University Diessen, Diessen, Hessen, Germany; Venatorx Pharmaceuticals, Malvern, Pennsylvania; Venatorx Pharmaceuticals, Malvern, Pennsylvania

## Abstract

**Background:**

Taniborbactam is an investigational β-lactamase inhibitor that restores cefepime (FEP) activity against FEP-, carbapenem-, and multidrug-resistant Enterobacterales and *Pseudomonas aeruginosa* producing serine- and metallo-β-lactamases. Cefepime-taniborbactam (FTB) was superior to meropenem (MEM) for the primary composite endpoint of microbiologic and clinical success at test of cure (TOC) in adults with cUTI in the CERTAIN-1 study (NCT03840148). We assessed outcomes in subsets defined by baseline pathogen resistance phenotype and genotype.

**Methods:**

Composite, microbiologic and clinical responses at TOC were assessed in the extended microbiologic intent-to-treat (microITT) population (patients with Enterobacterales and/or *P. aeruginosa* at ≥10^5^ CFU/mL in urine at study entry against which at least 1 study drug had activity [FTB MIC ≤16 µg/mL; MEM MIC ≤2 µg/mL (Enterobacterales) or ≤4 µg/mL (*P. aeruginosa*)]). Phenotypic subsets included FEP-, multidrug-, and/or carbapenem resistance. Genotypic subsets included carriage of ESBL, extended-spectrum AmpC, plasmidic AmpC, and/or carbapenemase genes.

**Results:**

For patients in the FTB treatment group with Enterobacterales at baseline, composite, microbiologic, and clinical success rates in resistant subsets were similar to or higher than those overall (Table). FTB achieved composite success in 7/8 patients with carbapenem-resistant Enterobacterales and in 8/9 patients with Enterobacterales producing a carbapenemase (5 OXA-48-group; 2 KPC-3, 2 NDM-1). FTB achieved composite success in 8/16 patients (clinical success in 13/16 patients) with *P. aeruginosa* overall; from 1 to 5 patients in the FTB group had *P. aeruginosa* in defined resistance categories.

**Figure:**
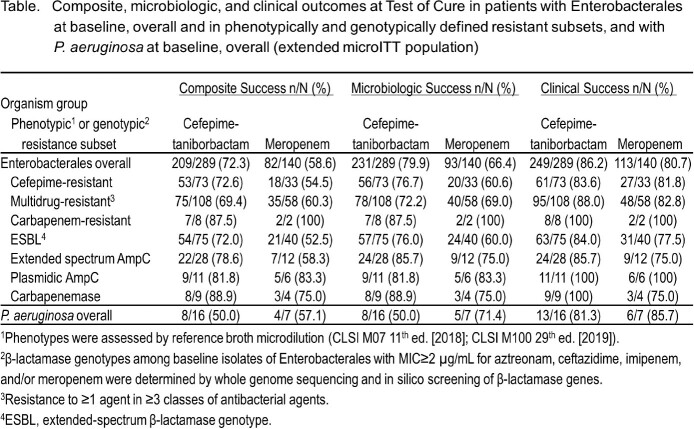

**Conclusion:**

FTB demonstrated efficacy in cUTI patients infected with cefepime-, multidrug-, and carbapenem-resistant pathogens and pathogens carrying ESBL, AmpC, and carbapenemase genes. Rates of success at TOC in each resistance subset were similar to or higher than those overall. These results are consistent with the ability of taniborbactam to restore cefepime activity against cefepime-, multidrug-, and carbapenem-resistant gram-negative pathogens producing serine and metallo-β-lactamases in nonclinical studies.

**Disclosures:**

**Greg Moeck, PhD**, Biomedical Advanced Research and Development Authority (BARDA): Grant/Research Support|Everest Medicines: Grant/Research Support|Global Antibiotic Research and Development Partnership (GARDP Foundation): Grant/Research Support|Venatorx Pharmaceuticals, Inc.: Grant/Research Support|Venatorx Pharmaceuticals, Inc.: Employee, stock options and shareholder **Leanne Gasink, MD, MSCE**, Biomedical Advanced Research and Development Authority (BARDA): Grant/Research Support|CSL Behring: Advisor/Consultant|CSL Behring: Consulting fees|Everest Medicines: Grant/Research Support|Evopint Biosciences: Advisor/Consultant|Evopint Biosciences: Consulting fees|Global Antibiotic Research and Development Partnership (GARDP Foundation): Grant/Research Support|LBG Consulting, LLC: Principal|Spero Therapeutics: Advisor/Consultant|Spero Therapeutics: Consulting fees|Venatorx Pharmaceuticals, Inc.: Advisor/Consultant|Venatorx Pharmaceuticals, Inc.: Grant/Research Support|Venatorx Pharmaceuticals, Inc.: Consulting fees|Vera Therapeutics: Advisor/Consultant|Vera Therapeutics: Consulting fees **Mary Beth Dorr, PhD**, Biomedical Advanced Research and Development Authority (BARDA): Grant/Research Support|Everest Medicines: Grant/Research Support|Global Antibiotic Research and Development Partnership (GARDP Foundation): Grant/Research Support|Merck and Co.: Shareholder|Pfizer: Shareholder|Venatorx Pharmaceuticals, Inc.: Grant/Research Support|Venatorx Pharmaceuticals, Inc.: Employee, stock options and shareholder **Hongzi Chen, PhD**, Biomedical Advanced Research and Development Authority (BARDA): Grant/Research Support|Everest Medicines: Grant/Research Support|Global Antibiotic Research and Development Partnership (GARDP Foundation): Grant/Research Support|Venatorx Pharmaceuticals, Inc.: Grant/Research Support|Venatorx Pharmaceuticals, Inc.: Employee|Venatorx Pharmaceuticals, Inc.: Stocks/Bonds **Rodrigo E. Mendes, PhD**, AbbVie: Grant/Research Support|Basilea: Grant/Research Support|Cipla: Grant/Research Support|Entasis: Grant/Research Support|GSK: Grant/Research Support|Paratek: Grant/Research Support|Pfizer: Grant/Research Support|Shionogi: Grant/Research Support **Florian Wagenlehner, MD**, Achaogen: Advisory Board member, study participation|Astellas: Honoraria|AstraZeneca: Honoraria|AstraZeneca: Advisory Board member|Biomedical Advanced Research and Development Authority (BARDA): Grant/Research Support|Bionorica: Honoraria|Bionorica: Meeting/travel support, study participation|Deutsches Zentrum für Infektionsforschung (DZIF): Study participation|Enteris BioPharma: Study participation|Everest Medicines: Grant/Research Support|German S3 guideline Urinary tract infections: Board Member|Glaxo Smith Kline: Advisor/Consultant|Glaxo Smith Kline: Honoraria|Glaxo Smith Kline: Consulting fees, meeting/travel support, advisory board member, principal investigator in a GSK-sponsored study|Global Antibiotic Research and Development Partnership (GARDP Foundation): Grant/Research Support|Guidelines European Association of Urology: Infections in Urology: Board Member|Helperby Therapeutics: Study participation|Janssen: Honoraria|Janssen: Advisory Board member|Klosterfrau: Honoraria|LeoPharma: Advisory Board member|MerLion: Advisory Board member|MIP Pharma: Honoraria|MSD: Advisory Board member|OM Pharma/Vifor Pharma: Advisory Board member, study participation|OM-Pharma: Honoraria|Pfizer: Honoraria|Pfizer: Advisory Board member|RosenPharma: Advisory Board member|Shionogi: Advisory Board member, study participation|Speaker research group German research foundation (DFG) Bacterial Renal Infections and Defense (FOR 5427): Study participation|Spero Therapeutics: Advisor/Consultant|Spero Therapeutics: Consulting fees|University Hospital Giessen and Marburg GmbH, and Justus Liebig University, Germany: Employee|Venatorx Pharmaceuticals, Inc.: Advisor/Consultant|Venatorx Pharmaceuticals, Inc.: Grant/Research Support|Venatorx Pharmaceuticals, Inc.: Consulting fees, Advisory Board member **Tim Henkel, MD, PhD**, Biomedical Advanced Research and Development Authority (BARDA): Grant/Research Support|Everest Medicines: Grant/Research Support|Global Antibiotic Research and Development Partnership (GARDP Foundation): Grant/Research Support|Venatorx Pharmaceuticals, Inc.: Advisor/Consultant|Venatorx Pharmaceuticals, Inc.: Employee, consulting fees, shareholder **Paul McGovern, MD**, Biomedical Advanced Research and Development Authority (BARDA): Grant/Research Support|Everest Medicines: Grant/Research Support|Global Antibiotic Research and Development Partnership (GARDP Foundation): Grant/Research Support|Paratek Pharmaceuticals: Shareholder|Venatorx Pharmaceuticals, Inc.: Grant/Research Support|Venatorx Pharmaceuticals, Inc.: Employee, stock options and shareholder

